# Supplementary material for: MetaRibo-Seq measures translation in microbiomes
Source: Nat Commun. 2020 Jun 29;11:3268. doi: 10.1038/s41467-020-17081-z (PMC7324362; doi:10.1038/s41467-020-17081-z)
Supplement: Supplementary file 10 — Supplementary Data 7 [file 41467_2020_17081_MOESM10_ESM.zip › File2/Confidence_VeryHigh_Taxonomy/319789_out.krona.html]

Javascript must be enabled to view this page.

members
magnitude
magnitudeUnassigned
count
unassigned
taxon
rank

319789\_out

41

superkingdom
41
2

976
41
phylum

41
class
200643

171549
41
order

family
31
2005525

genus
31
375288

823

SRS011134\_contig\_number\_27142SRS011239\_contig\_number\_23786SRS014313\_contig\_number\_1029SRS015663\_contig\_number\_28151SRS016095\_contig\_number\_14677SRS016267\_contig\_number\_8734SRS018656\_contig\_number\_9706SRS019787\_contig\_number\_10576SRS043768\_contig\_number\_16011SRS045195\_contig\_number\_3976SRS046712\_contig\_number\_745SRS052027\_contig\_number\_19529SRS053649\_contig\_number\_21911SRS058723\_contig\_number\_6916SRS064645\_contig\_number\_2074SRS076929\_contig\_number\_26887SRS077194\_contig\_number\_22632SRS077335\_contig\_number\_1499SRS078242\_contig\_number\_21275SRS098061\_contig\_number\_3593SRS100021\_contig\_number\_11782SRS1041095\_contig\_number\_5187SRS104693\_contig\_number\_3597SRS143876\_contig\_number\_36223SRS146764\_contig\_number\_contig-100\_104.201478SRS146813\_contig\_number\_4784SRS147272\_contig\_number\_254SRS147377\_contig\_number\_3532SRS148874\_contig\_number\_4610SRS893288\_contig\_number\_4582
30
species

1
species
563193

SRS056273\_contig\_number\_23192

815
4
family

1
genus

SRS077086\_contig\_number\_3707
4
816

species
1
310297

SRS893285\_contig\_number\_3146

species
2
821

SRS016056\_contig\_number\_5847SRS1055049\_contig\_number\_10331

171551
family
3

genus
3
836

3
species

SRS019445\_contig\_number\_20981SRS043411\_contig\_number\_13934SRS050299\_contig\_number\_contig-100\_31.81677
658663

family
2
171552

genus
2
838

1
species
363265

SRS1041116\_contig\_number\_5028


SRS049896\_contig\_number\_604
1262929
1
species

1
family
171550

1
genus
239759

1118061

SRS104975\_contig\_number\_12634
1
species
